
JOURNAL INFORMATION
==============================
NLM Title Abbreviation: Behav Neurol
Journal ID: Behav Neurol
NLM Journal ID: 8914585
Journal ID: BN

Behavioural Neurology

ISSN: 0953-4180
EISSN: 1875-8584
Publisher: Wiley

ARTICLE INFORMATION
==============================
PMCID: PMC5215698
PMID: 22713414
DOI: 10.3233/BEN-2012-129002
Article ID: 271815
Article version: 1
Subjects: Other

Word-Superiority in Pure Alexia

Starrfelt R. 1 *
Gerlach C. 2
Habekost T. 1
Leff A. P. 3
1 Center for Visual Cognition Department of Psychology University of Copenhagen Copenhagen Denmark
2 Department of Psychology University of Southern Denmark Odense Denmark
3 Institute of Neurology and National Hospital for Neurology and Neurosurgery University College London London UK
*R. Starrfelt: randi.starrfelt@psy.ku.dk
Print publication date: 2013
Volume: 26
Issue: 3
First page: 167
Last page: 169
Received 2012 May 21; Accepted 2012 May 21
Copyright: Copyright © 2013 Hindawi Publishing Corporation and the authors.
Copyright year: 2013
License: This is an open access article distributed under the Creative Commons Attribution License, which permits unrestricted use, distribution, and reproduction in any medium, provided the original work is properly cited.
License URL: https://creativecommons.org/licenses/by/3.0/

Keywords: Pure alexia, word reading, visual field defect

Funding: http://dx.doi.org/10.13039/501100001734 University of Copenhagen

==============================
Behavioural Neurology 26 (2013) 167–169
DOI 10.3233/BEN-2012-129002
IOS Press

### Word-superiority in pure alexia

R. Starrfelt[a,*], C. Gerlach[b], T. Habekost[a] and A.P. Leff[c]
[a]*Center for Visual Cognition, Department of Psychology, University of Copenhagen, Copenhagen, Denmark*
[b]*Department of Psychology, University of Southern Denmark, Odense, Denmark*
[c]*Institute of Neurology and National Hospital for Neurology and Neurosurgery, University College London, London, UK*

Keywords: Pure alexia, word reading, visual field defect

### 1. Introduction

Pure alexia is an acquired reading disorder where patients lose the ability to read fast and fluently, but are commonly able to identify words letter-by-letter. This is evidenced by a pronounced word-length effect in reading, where reaction times (RTs) increase linearly with word length. The severity of the reading deficit in pure alexia has been reported to be systematically related to performance on lexical and semantic decision tasks [1] and to visuoperceptual deficits [2]. Lesions to the suggested 'visual word form area' (VWFA) located in the left mid-fusiform gyrus seem to be of particular importance in causing pure alexia [3,4].

In the current study, we examine the *word superiority effect* (*WSE*) in four pure alexic patients. The WSE refers to the phenomenon that normal readers are better at identifying letters embedded in words than in letter strings [5]. The effect is typically found in experiments where stimuli are presented briefly and then masked, followed by either a forced choice or free report task. There are a few case studies of the word-superiority effect in pure alexia (e.g. [6]) but findings so far have been contradictory. There have been no systematic attempts at linking performance in WSE experiments to visuoperceptual abilities or severity in pure alexia.

*Corresponding author: R. Starrfelt, Center for Visual Cognition, Department of Psychology, Copenhagen University, O. Farimagsgade 2a, DK-1353 Copenhagen K, Denmark. E-mail: randi.starrfelt @psy.ku.dk.

### 2. Materials and methods

### *2.1. Subjects*

Four patients with pure alexia and six control participants. Each subject gave informed consent to participate in the study that was approved by an NHS local research ethics committee (Royal Free Hospital).

### *2.2. Neuropsychological data*

All patients have pure alexia, with elevated RTs in single word reading, a significant effect of word length on RTs, and normal performance on writing tests. All patients showed reduced visual processing speed for singly presented letters and digits, and reduced visual apprehension span for unrelated letters and digits presented simultaneously. Auditory span was within normal range. One patient (BA) had a complete right sided hemianopia, two patients had partial right hemianopias, while one patient (TJ) had full visual fields. For detailed descriptions and tests, see [7].

### *2.3. Imaging*

All four patients have lesions to the posterior left hemisphere including the mid fusiform gyrus (the VWFA; for further description and images, see [7]).

ISSN 0953-4180/13/$27.50 © 2013 – IOS Press and the authors. All rights reserved

Table 1
Mean correct scores (max = 6) for words and nonwords for patients and controls in the WSE experiment, and the mean word superiority effect. For comparison, mean reading RTs (in msec) for words of 3–7 letters, as well as the estimated word length effect (in msec/letter) are presented at the bottom row (from [7])

| | TJ | JT | BA | JH | Ctrl (SD) |
|---|---|---|---|---|---|
| *WSE experiment* | | | | | |
| *Words* | 4.25** | 2.72** | 1.83** | 2.75** | 5.93 (0.04) |
| *Nonwords* | 1.76** | 1.83** | 1.19** | 1.96** | 4.13 (0.34) |
| *WSE* | 2.49 | 0.89* | 0.64* | 0.79* | 1.8 (0.38) |
| *Single word reading* | | | | | |
| *RTs* | 1388** | 912** | 1351** | 1562** | 438 (43) |
| *Word length effect* | 212** | 176** | 201** | 146** | 1.74 (1.81) |

$^{**}p < 0.001$; $^{*}p < 0.05$ (one-tailed) Crawford and Howell's test [9].

### 2.4. Word superiority experiment

We investigated visual apprehension span for letters in words vs. unrelated letter-strings. The experiment was conducted in a semi-darkened room, and subjects were seated approximately 100 cm from a 19" CRT monitor.

#### 2.4.1. Stimuli

100 real words of five or seven letters were selected from [[8]; appendix B]. Two lists were created, both containing 25 five letter, and 25 seven letter words. The lists were matched for frequency and orthographic neighborhood size. For each list, we jumbled the letters of the words to create 50 nonwords of 5 and 7 letters, keeping summed letter confusability constant for words and nonwords.

#### 2.4.2. Procedure

Subjects fixated a central cross, and indicated verbally when they were ready. The experimenter initiated a trial where a stimulus (word or letter string) was presented for either 100 ms postmasked or 200 ms unmasked. Masked trials were instantly followed by a pattern mask for 500 ms. Stimuli were presented in 36 point Times New Roman, white on black. A total of 200 trials were performed, divided over two sessions. Trials were randomly presented for each participant, but the same words were presented in each condition (masked/unmasked) for all participants.

#### 2.4.3. Task

Subjects were asked to report as many letters as possible from the display, but refrain from naming words. This has been shown to yield the most reliable measures of WSE [5]. Subjects were asked to name the letters from left to right. They were told that both words and nonwords would be presented.

#### 2.4.4. Scores

We computed a score for correctly reported letters in correct position.

### 3. Results

See Table 1 for summary of results. All subjects show a word-superiority effect in letter report: they report significantly more letters from words than from nonwords. At the same time, all patients report significantly fewer letters in all conditions compared to controls (Crawford and Howell's test [9]). The size of the WSE differs between patients. While three patients show WSE's of a significantly smaller magnitude than controls, patient TJ shows a WSE on the same level as the controls. TJ's reading RTs were on the same level as the other patients, and there is no correlation between the magnitude of the patients' WSE and reading RTs. Nor is there any systematic relationship between the magnitude of the WSE and the patients' degree of reduction in visual apprehension span or processing speed for unrelated letters (reported in [7]). There is, however, a significant negative correlation between the size of the patients' field defects, and the magnitude of their WSE (Spearman's rho $p < 0.01$).

### 4. Conclusions

A significant word superiority effect was found in four patients with lesions affecting the visual word form area. For three patients, however, this effect was smaller than for controls. There was no systematic relationship between the patients reading performance and the magnitude of the WSE. There was, however, a correlation between the size of the patients' visual field defect, and the magnitude of their WSE. The one patient with normal WSE also had full visual fields, but was still impaired in reading to the same degree as the other patients.

This suggests that the presence and size of a visual field defect may affect single word processing in pure alexia. Previous investigations have compared eye movement patterns of pure alexic patients with patients suffering from hemianopic alexia, but to our knowledge, there have been no investigation of the direct contribution of visual field defects to single word reading in pure alexia. Our results suggest that this relationship should be examined further, and in depth studies of pure alexic patients without hemianopia would be particularly interesting in this respect.

### Acknowledgments

This work was supported by University of Copenhagen's Center of Excellence Program (Grant to RS/ Center for Visual Cognition) and Fakutsi.

### References

[1] D.J. Roberts, M.A. Lambon Ralph and A.M. Woollams, When does less yield more? The impact of severity upon implicit recognition in pure alexia, *Neuropsychologia* **48**(9) (2010), 2437–2446.

[2] R.H. Mycroft, M. Behrmann and J. Kay, Visuoperceptual deficits in letter-by-letter reading? *Neuropsychologia* **47** (2009), 1733–1744.

[3] A.P. Leff, G. Spitsyna, G.T. Plant and R.J. Wise, Structural anatomy of pure and hemianopic alexia, *Journal of Neurology, Neurosurgery and Psychiatry* **77** (2006), 1004–1007.

[4] T. Pflugshaupt, K. Gutbrod, P. Wurtz, R. von Wartburg, T. Nyffeler, B. de Haan et al., About the role of visual field defects in pure alexia, *Brain* **132** (2009), 1907–1917.

[5] J.L. McClelland and J.C. Johnston, The role of familiar units in perception of words and nonwords, *Perception and Psychophysics* **22** (1977), 249–261.

[6] R. Starrfelt, T. Habekost and C. Gerlach, Visual processing in pure alexia: A case study, *Cortex* **46** (2010), 242–255.

[7] R. Starrfelt, T. Habekost and A.P. Leff, Too little, too late: reduced visual span and speed characterize pure alexia, *Cerebral Cortex* **19** (2009), 2880–2890.

[8] K. Osswald, G.W. Humphreys and A. Olson, Words are more than the sum of their parts: Evidence for detrimental effects of word-level information in alexia, *Cognitive Neuropsychology* **19** (2002), 675–695.

[9] J.R. Crawford and D.C. Howell, Comparing an individual's test score against norms derived from small samples, *The Clinical Neuropsychologist* **12** (1998), 482–486.
